# First Report of Respiratory Infection Caused by Multidrug-Resistant *Escherichia coli* in an Ostrich in Romania

**DOI:** 10.3390/antibiotics14040354

**Published:** 2025-03-31

**Authors:** Vlad Iorgoni, Ionica Iancu, Ionela Popa, Alexandru Gligor, Gabriel Orghici, Bogdan Sicoe, Cristian Dreghiciu, David Purec, Paula Nistor, Bogdan Florea, Viorel Herman

**Affiliations:** 1 Department of Infectious Diseases and Preventive Medicine, Faculty of Veterinary Medicine, University of Life Sciences “King Mihai I” from Timişoara, 300645 Timişoara, Romania; vlad.iorgoni@usvt.ro (V.I.); alexandru.gligor@usvt.ro (A.G.); david.purec.fmv@usvt.ro (D.P.); paula.nistor@usvt.ro (P.N.); viorel.herman@fmvt.ro (V.H.); 2 Department of Semiology, Faculty of Veterinary Medicine, University of Life Sciences “King Mihai I” from Timişoara, 300645 Timişoara, Romania; ionela.popa@usvt.ro; 3 Department of Veterinary Emergencies, Faculty of Veterinary Medicine, University of Life Sciences “King Mihai I” from Timisoara, 300645 Timişoara, Romania; gabriel.orghici@usvt.ro; 4 Department of Radiology and Imaging, Faculty of Veterinary Medicine, University of Life Sciences “King Mihai I” from Timisoara, 300645 Timişoara, Romania; bogdan.sicoe@usvt.ro; 5 Department of Parasitology, University of Life Sciences “King Mihai I” from Timisoara, 300645 Timişoara, Romania; cristian.dreghiciu@usvt.ro; 6 Department of Internal Medicine, University of Life Sciences “King Mihai I” from Timisoara, 300645 Timişoara, Romania; bogdan-alexandru.florea.fmv@usvt.ro

**Keywords:** ostrich, *Escherichia coli*, respiratory infection, antimicrobial resistance

## Abstract

Introduction: Ostrich farming is increasingly recognized for its economic potential but poses significant health challenges due to the risk of pathogen transmission, including multidrug-resistant *Escherichia coli*. Case study: This study reports a case of a four-month-old female ostrich from western Romania presenting with severe respiratory and digestive infections, progressing to septicemia and death. A post-mortem examination revealed extensive mucus in the trachea, pulmonary congestion, hemorrhagic enteritis, and approximately 1250 g of metal objects in the ventriculus. Pure cultures of *E. coli* were isolated from the lungs and bone marrow and identified via MALDI-TOF MS. The strain exhibited multidrug resistance to several antibiotics, including enrofloxacin, doxycycline, and amoxicillin, highlighting the critical issue of antimicrobial resistance in veterinary medicine. Discussions: This case underscores the need for enhanced management practices in ostrich farming to mitigate environmental and pathogenic risks, as well as the urgency of developing alternative strategies for controlling resistant bacterial infections in avian species. Conclusions: This case highlights the need for alternative treatments and stricter antimicrobial stewardship to combat multidrug-resistant *E. coli* in ostriches.

## 1. Introduction

The emergence of multidrug-resistant (MDR) bacteria represents a significant public health threat, as highlighted by the World Health Organization. The increasing prevalence of infections caused by MDR pathogens, coupled with the limited availability of effective treatments, is expected to lead to higher mortality rates among both humans and animals affected by infectious diseases. Multidrug resistance is typically characterized by resistance to antibiotics from at least three distinct classes [1,2,3,4,5].

Ostrich farming has emerged as a growing agricultural practice worldwide, driven by the demand for high-quality products such as meat, leather, feathers, and oil. However, this industry faces significant challenges, particularly regarding the health status of imported ostriches, which may serve as reservoirs for exotic pathogens, including both bacterial and viral agents. This poses potential threats to domestic poultry populations, especially as the interaction between ostriches and poultry increases. Among these concerns, respiratory diseases caused by pathogenic strains of *Escherichia coli* have been identified in ostriches, yet the virulence factors and cross-species pathogenic potential of these strains remain poorly understood [6,7,8,9,10].

Ostrich farming in Romania is a growing niche industry, with increasing interest due to the high value of ostrich meat, leather, and feathers. Although production remains limited, it is supported by regional markets in neighboring countries, which also have small-scale ostrich farms primarily focused on meat production. In Romania, the situation has also been marked by increasing concerns regarding antimicrobial resistance, particularly in *E. coli* strains isolated from farmed ostriches. Studies have shown that these strains exhibit significant resistance to common antimicrobials, highlighting the need for improved management and surveillance practices. Challenges in this industry include high production costs, limited consumer awareness, and the need for specialized management practices [7,11].

*E. coli*, a primary etiological agent of colibacillosis, is responsible for respiratory infections, septicemia, and systemic issues in poultry, leading to considerable economic losses. Key virulence factors, including adhesins, serum resistance, and toxin production, enhance host colonization and immune evasion. Despite ongoing research, effective and broadly protective vaccines are still under development, leaving antimicrobial treatments, such as florfenicol, apramycin, and danofloxacin, as the primary control measures. However, the widespread use of antibiotics has raised concerns about antimicrobial resistance and heightened consumer demand for antibiotic-free poultry products, necessitating alternative control strategies [10,12,13,14,15,16,17].

Environmental conditions in ostrich farming play a pivotal role in pathogen trans-mission and disease development. Poor hygiene in hatcheries, microbial contamination of hatching eggs, and inadequate housing conditions contribute to the spread of pathogens, including *E. coli* and *Salmonella* spp. Furthermore, structural factors, such as the flooring and drainage design, significantly influence the microbial load, with direct implications for animal health and product safety [7,13,17,18,19,20,21].

Inadequate management practices, such as poor biosecurity, improper nutrition, and unsanitary living conditions, are significant risk factors for disease onset in farmed ostriches. Stress factors, like overcrowding and improper handling, can also weaken their immune system, increasing their susceptibility to infections. Beyond *Escherichia coli*, other pathogens, such as *Pseudomonas aeruginosa*, *Salmonella* spp., *Staphylococcus aureus*, and *Campylobacter jejuni*, have been implicated in septicemic infections in ostriches. Additionally, diseases, such as Crimean-Congo hemorrhagic fever, spongiform encephalopathy, and velogenic *Newcastle disease virus* (NDV), pose significant risks to ostrich populations. While *Salmonella typhimurium* can cause mortality in young ostriches, diseases like *Mycoplasma* spp., haemorrhagic septicemia, and fowl cholera remain common challenges, especially in poorly managed environments. The lack of comprehensive diagnostic tools further complicates disease management, underscoring the importance of improved surveillance and disease prevention strategies in both farmed and zoo-housed ostriches [7,18,19,20].

The respiratory tract microbiota in birds has gained attention for its role in immune modulation and pathogen defense. However, its composition and influence on respiratory diseases, particularly those caused by *E. coli*, remain underexplored. The increasing prevalence of antimicrobial resistance and horizontal gene transfer further complicates the effective management of respiratory infections [4,6,7,22].

Avian pathogenic *E. coli* (APEC) is a major pathogen in poultry, causing extraintestinal infections such as respiratory disease, septicemia, and pericarditis. APEC strains share genetic similarities with human *E. coli* strains involved in urinary tract infections and sepsis. Given the zoonotic potential of APEC, there is a growing concern about the transmission of resistant strains from poultry to humans, particularly through contaminated poultry products [23,24].

In this study, we present the case of a four-month-old ostrich from a farm in western Romania that presented with severe clinical signs, including respiratory and digestive infections, which progressed to septicemia and death. A post-mortem analysis identified *Escherichia coli* as the sole pathogen, isolated from lung and bone marrow samples. The strain exhibited multidrug resistance to several antibiotics, posing significant therapeutic challenges and highlighting the critical issue of antimicrobial resistance in veterinary medicine.

## 2. Case Study

In this study, we present the case of a young female ostrich that was presented to the Emergency Service of the Faculty of Veterinary Medicine in Timișoara, Romania, after being raised in suboptimal conditions at a farm in the western region of the country. The farm housed seven other ostriches, alongside other birds, and no antibiotics had been administered to the flock prior to the onset of illness. The affected animal had ingested various metal objects weighing approximately 1250 g. The ostrich exhibited symptoms of a respiratory and digestive infection, which eventually led to septicemia and death. The case was complicated by the infection of a multidrug-resistant strain of *Escherichia coli*.

The owner reported a progressive decline in the ostrich’s condition, characterized by lethargy, reduced food intake, and weight loss. Upon arrival at the veterinary clinic, the ostrich was severely debilitated, unable to lift its head, and exhibited marked lethargy. Temperature measurements indicated severe hypothermia, with the thermometer unable to register a temperature. The mucous membranes were pale, and the oral cavity was filled with viscous mucus, obstructing respiration. Immediate stabilization measures included warming, rehydration, and the insertion of an intravenous catheter. Shortly after, the ostrich suffered cardiorespiratory arrest. Emergency resuscitation, including cardiac massage, artificial ventilation, and adrenaline administration (0.03 mg/kg in two doses), was performed [25]. After 20 min, the ostrich’s heart resumed regular beats, it began breathing independently, and pupillary reflexes were restored. The bird was subsequently transferred to intensive care for continued monitoring, and enrolfloxacin was administered because of the suspicion of a respiratory infection.

However, approximately six hours later, the ostrich experienced another cardiorespiratory arrest, which could not be reversed despite further resuscitation efforts. A post-mortem examination was performed to identify the cause of death.

**Figure 1:**
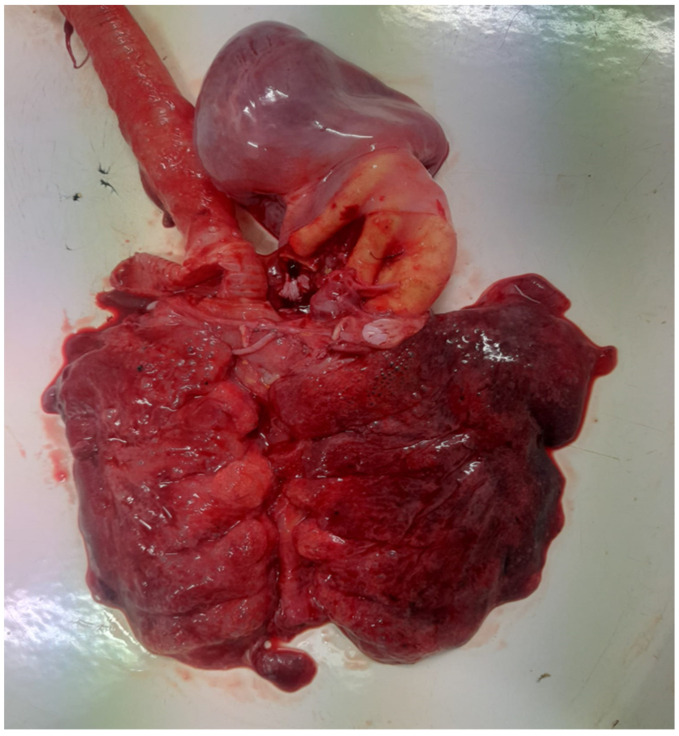
Congested and hemorrhagic lungs with tracheal hyperemia.

The post-mortem examination revealed significant pathological changes consistent with septicemia. The trachea contained a substantial amount of mucus and exhibited severe congestion and hemorrhages. The lungs displayed a diffuse pattern of congestion (Figure 1) and petechial hemorrhages, which were not restricted to dependent regions, ruling out post-mortem artifacts. Right-sided cardiac dilatation and opacity and increased pericardial fluid were also observed. Additionally, the liver was enlarged, congested, and friable, while the spleen was markedly enlarged and hyperemic. The kidneys appeared swollen and congested, with notable vascular engorgement. Hemorrhagic enteritis was present and the pancreas was enlarged with diffuse congestion. The small intestine, particularly the duodenum, jejunum, and ileum, exhibited severe congestion, with diffuse mucosal hyperemia and petechial hemorrhages. Fibrinous to fibrino-hemorrhagic exudate was present in moderate to abundant amounts, adhering to the intestinal mucosa. Cecal involvement was also noted, with areas of caseous exudate. The intestinal serosa displayed marked vascular engorgement. A thickening and opacity of the air sacs further indicated respiratory involvement. The necropsy was conducted within hours after death, minimizing the likelihood of hypostatic changes and supporting the interpretation of these findings as genuine pathological lesions associated with septicemia. Notably, approximately 1250 g of metal objects were found in the ventriculus. Upon examination of the ventriculus content, approximately 30 foreign metallic objects were identified. However, none of these objects had sharp edges or pointed structures capable of disrupting or damaging the inner surface of the organ. The mucosa remained intact, with no visible lesions or perforations, indicating that these foreign bodies did not pose a risk for peritonitis. Samples were collected from the lungs, intestines, and bone marrow (femur), and cultures were performed.

To rule out heavy metal intoxication due to the presence of foreign metallic objects in the ventriculus, toxicology tests were performed. Specific tests, including blood lead testing, atomic absorption spectroscopy (AAS), and inductively coupled plasma mass spectrometry (ICP-MS) for lead, cadmium, and mercury levels in blood and tissues was conducted. The results of these tests confirmed that heavy metal intoxication was not involved in this case [26,27,28].

**Figure 2:**
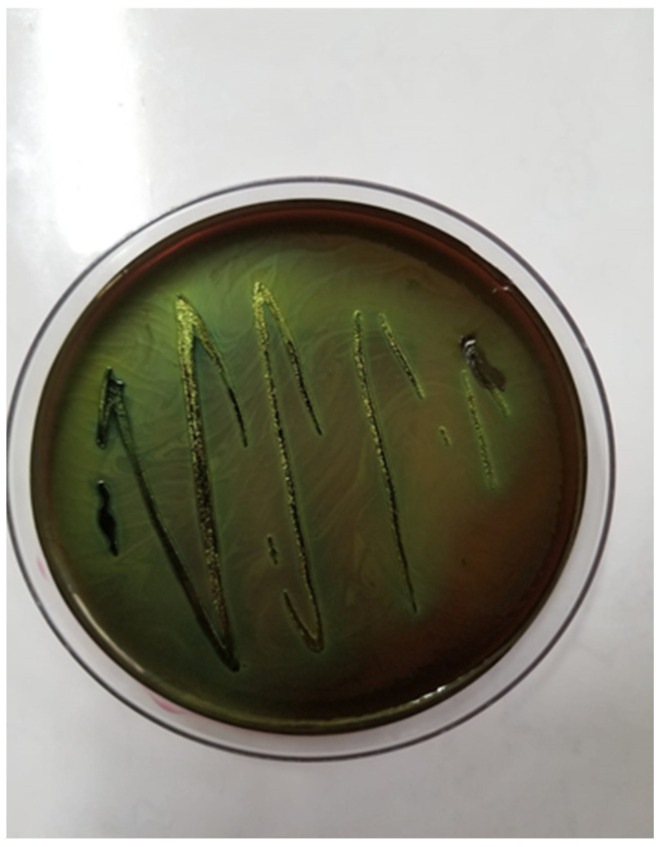
*Escherichia coli* colonies on EMB agar with a green metallic sheen.

**Figure 3:**
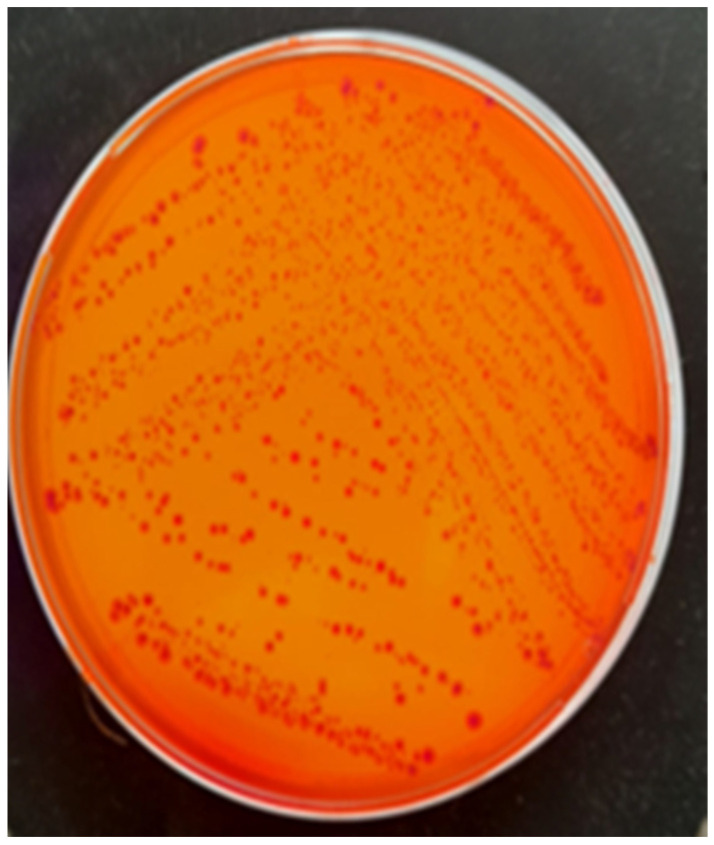
*Escherichia coli* colonies grown on Congo red agar, exhibiting Congo red binding.

Bacterial cultures were performed on Columbia agar with 5% sheep blood and MacConkey agar, followed by selective and differential media, such as eosin methylene blue (EMB) agar (Figure 2) and chromogenic agar. These media allowed the identification of colonies with characteristic morphology. The cultures were incubated at 37 °C for 24 h under aerobic conditions. The only pathogen isolated from all tissues was identified as *E. coli*, confirming its role in septicemia. A further characterization of the pathogenic potential of the isolate was performed using Congo red agar, revealing Congo red binding (Figure 3), a feature associated with increased virulence in pathogenic *E. coli* strains.

Molecular analysis of the *E. coli* strain isolated from the ostrich confirmed its pathogenic potential, revealing key virulence genes. PCR identified *ompA* (invasion), *fimH* (adhesion), and *iss* (immune evasion). These findings highlight the strain’s ability to colonize, invade, and cause systemic infection, supporting its role in respiratory disease and septicemia [29,30].

Pure bacterial cultures were obtained from both lung tissue and femoral bone marrow samples, confirming systemic dissemination of the pathogen. The strain was identified as *E. coli* using matrix-assisted desorption–ionization time-of-flight mass spectrometry (MALDI-TOF MS). Additionally, biochemical identification was performed using the API 20E test, further confirming the presence of *E. coli*.

Quality control procedures were rigorously followed to ensure the accuracy and reliability of the susceptibility testing. For this purpose, the ATCC 25922 strain, *E. coli* strain, was used as a standard reference strain to validate the testing process. The results obtained from the quality control strain were carefully monitored and evaluated to ensure they fell within the established quality control ranges. This confirmation that the *E. coli* ATCC 25922 strain’s susceptibility profile remained within acceptable limits, with an identity similarity index of 2.15, provided assurance that the testing methods were accurate and consistent, thereby validating the results obtained for the experimental strains.

The antimicrobial susceptibility testing of the isolate was performed using the VITEK 2 automated system (bioMérieux, Marcy-l’Étoile, France), according to the manufacturer’s instructions. The minimum inhibitory concentrations (MICs) of the tested antibiotics were as follows: fluoroquinolones (enrofloxacin: ≥8 µg/mL), tetracyclines (doxycycline: ≥16 µg/mL, tetracycline: ≥16 µg/mL), beta-lactams (penicillin: ≥2 µg/mL, ampicillin: ≥8 µg/mL, amoxicillin: ≥8 µg/mL, ceftazidime: ≥16 µg/mL), sulfonamides (co-trimoxazole: ≥320 µg/mL), and macrolides (erythromycin: ≥8 µg/mL). The isolate demonstrated resistance to all of these antibiotic classes. However, it exhibited susceptibility to aminoglycosides (gentamicin: ≤1 µg/mL) [31].

Multidrug resistance was defined as resistance to at least one agent in three or more antimicrobial categories, following widely accepted international criteria. This definition allows for a standardized classification of bacterial resistance patterns, facilitating comparisons across studies and ensuring consistency in the assessment of antimicrobial susceptibility profiles [32].

The *E. coli* strain exhibited resistance to fluoroquinolones (enrofloxacin), tetracyclines (doxycycline, tetracycline), beta-lactams (penicillin, ampicillin, amoxicillin, ceftazidime), sulfonamides (co-trimoxazole), and macrolides (erythromycin). However, it remained susceptible only to aminoglycosides (gentamicin). The antibiogram results indicated a resistant profile, which correlated with the severity of the infection and the lack of therapeutic success. Despite the administration of enrofloxacin, the treatment proved ineffective, highlighting the pathogen’s resistance.

## 3. Discussion

This case highlights the severe impact of multidrug-resistant *E. coli* in ostriches, leading to pulmonary infection and septicemia. The findings show that ostriches raised under suboptimal conditions, such as poor hygiene and high stress, are particularly vulnerable to such infections. The development of antibiotic-resistant strains make treatment failure more likely and increasing the risk of mortality.

The isolation of *E. coli* from multiple organs, including lung tissue and femoral bone marrow, confirms the systemic dissemination of the pathogen, leading to septicemia and fatal outcomes. The strain exhibited resistance to several commonly used antibiotics, including enrofloxacin, doxycycline, penicillin, and ceftazidime, with susceptibility observed only to gentamicin. The presence of Congo red binding further indicated its high virulence potential. These results emphasize the need for rigorous antimicrobial stewardship and improved management practices in ostrich farming to mitigate the risks of bacterial infections and enhance disease prevention strategies.

A study on bacterial enteritis in ostrich chicks in the Western Cape Province of South Africa identified enteropathogenic and enterotoxigenic *E. coli* as causative agents, highlighting the role of environmental factors and stress in disease development [33].

Research on bacterial, fungal, and parasitic infections in ostriches notes that *Pasteurella multocida* can cause air sac infections, while *E. coli* is associated with colibacillosis, emphasizing the impact of husbandry practices on health outcomes [34].

A case report on *Enterococcus casseliflavus* infection in a captive southern cassowary describes multiorgan involvement, including endocarditis and cholangiohepatitis, demonstrating the potential severity of bacterial infections in ratites [35].

Previous reports have highlighted the increasing prevalence of bacterial infections, particularly those caused by multidrug-resistant *E. coli* in ostriches and other ratites, often associated with suboptimal farming conditions. It was noted that poor management practices, including inadequate hygiene and nutrition, contribute to the susceptibility of ostriches to various bacterial pathogens, including *E. coli* and *Clostridium perfringens*. Similarly, *E. coli* was identified as a major pathogen in ostrich chicks suffering from enteritis, with multidrug-resistant strains being a significant concern for treatment failure. The current case report aligns with these findings, particularly the isolation of multidrug-resistant *E. coli* from the lungs and bone marrow of an ostrich, emphasizing the growing challenge of antimicrobial resistance in avian species. Furthermore, the presence of foreign metallic objects in the ventriculus adds a layer of complexity to the diagnosis, with the potential for exacerbating the infection risk, as previously observed in cases of foreign body ingestion in avian species. These cases underscore the importance of improving farming conditions and implementing strict biosecurity measures to reduce pathogen transmission and the emergence of resistant bacterial strains [33,34,35,36,37,38].

Avian colibacillosis caused by *E. coli* leads to respiratory infections and septicemia in poultry, with common lesions such as airsacculitis. Studies in Egypt and other regions report high *E. coli* prevalence, which are linked to respiratory distress and sudden deaths in broilers [31,32]. Similar manifestations have been observed in ostriches, with fibrinous lesions and septicemia at post-mortem examination [38,39].

In poultry, *E. coli* is a major cause of economic losses, being implicated in colibacillosis, airsacculitis, salpingitis, and cellulitis. In humans, *E. coli*, is the leading cause of urinary tract infections, while neonatal meningitis-associated *E. coli* and sepsis-causing *E. coli* are linked to severe diseases, including neonatal meningitis and septicemia [39,40].

The presence of *E. coli* and *Salmonella* on the outer shell surface and within the con-tents of ostrich eggs highlights potential risks associated with egg contamination. The detection of *Salmonella* serovars other than *Salmonella* Enteritidis and *S. typhimurium*, along with the identification of *E. coli*, raises concerns regarding public health implications, as these pathogens have been associated with foodborne illness. The high incidence of microbial contamination observed in this study is consistent with previous findings in ostrich eggs and emphasizes the role of environmental factors and management practices in pathogen transmission. Given the potential for bacterial transfer to humans through direct contact or consumption of contaminated eggs, strict biosecurity measures, routine microbiological monitoring, and appropriate handling practices are essential to minimize the risk of zoonotic transmission [40,41].

In a 2020 study from China, researchers investigated antimicrobial resistance in *E. coli* isolates from chicken respiratory tracts, highlighting alarming resistance rates to florfenicol, apramycin, and danofloxacin. Given the high resistance rates observed, particularly for florfenicol and danofloxacin, there is an urgent need to reassess treatment regimens and implement stringent antimicrobial stewardship practices. The study high-lights the potential risk of treatment failure and the importance of routine resistance monitoring in veterinary medicine, especially for non-domesticated avian species, such as ostriches, which are increasingly raised in intensive farming systems [13].

In a study from 2016 from Slovakia, coliform bacteria were isolated from ostrich fecal samples across three age groups to assess their prevalence, identification, hemolytic properties, and antimicrobial resistance. MALDI-TOF MS identified *E. coli* as the predominant species, with some strains exhibiting β-hemolysis. Antibiotic susceptibility testing revealed a high resistance to penicillin and erythromycin, a moderate resistance to tetracycline and chloramphenicol, and a low resistance to gentamicin and cefalotin. Susceptibility testing to enterocins showed inhibitory effects, particularly from *Enterococcus faeciums* trains, suggesting potential antimicrobial alternatives. These findings highlight the presence of resistant *E. coli* in ostrich farming, underscoring the need for antimicrobial stewardship and further research into probiotic interventions [42].

A study from Bangladesh investigated the molecular characteristics, virulence genes, and antimicrobial resistance profiles of 392 *E. coli* isolates from six poultry farms in Bangladesh. A Congo red binding assay confirmed the pathogenic potential of 174 APEC isolates, which were classified into ten distinct genotypes using RAPD, BOX-PCR, and ERIC-PCR. Phylogenetic analysis revealed that the APEC isolates predominantly belonged to phylotypes B2 and A1, with B2 and D2 being significantly associated with colibacillosis. Most isolates (75–100%) harbored key virulence genes (ial, fimH, crl, papC, and cjrC) and 81.71% formed biofilms. Additionally, the isolates exhibited high antimicrobial resistance, with 100% resistance to at least three antibiotics. This study highlights the dominance of B2 and A1 phylotypes in Bangladesh and emphasizes the need for alternative antimicrobial strategies to combat antibiotic resistance in poultry farming [43].

A study was conducted on 42 *E. coli* strains isolated from broilers and poultry facilities in western Romania between 2010 and 2011 to assess the prevalence of avian pathogenic *E. coli* (APEC). Multiplex PCR was used to detect three virulence genes associated with APEC strains. The *OmpA* gene, responsible for bacterial attachment, was present in 18 strains, while the *iss* gene, which encodes a protein that promotes complement resistance and facilitates colonization, was found in 17 strains. The *fimH* gene, encoding type 1 fimbriae, was detected in 19 strains. All isolates exhibited typical *E. coli* biochemical characteristics and were found to be resistant to multiple antibiotics, including lincomycin, spectinomycin, neomycin, tetracycline, erythromycin, doxycycline, and enrofloxacin. Congo red binding was observed in correlation with the presence of these genes, serving as a phenotypic marker for avian-origin *E. coli* strains and aiding in the identification of the APEC pathotype [44].

Bacteriophages showed potential in protecting broiler chickens from *E. coli* respiratory infections when administered directly at the site of infection, with significant reductions in mortality. The mortality dropped from 80% to 25% and 5% when *E. coli* was mixed with 10^3^ or 10^6^ pfu/mL of bacteriophage, respectively. However, when bacteriophages were added to drinking water, no protection was observed. These findings suggest that bacteriophage therapy could be a promising alternative to antibiotics for poultry, particularly when applied locally to the infection site [45].

The management of *E. coli* infections often involves antibiotics, like cefquinome, though increasing antimicrobial resistance poses a challenge [32]. Vaccination with live attenuated *E. coli* strains has shown promise in reducing mortality in poultry [46], suggesting potential benefits for ostrich treatment. Additionally, bacteriophage therapy could offer an alternative to antibiotics, as seen in poultry studies, warranting further exploration in ostrich medicine [38,39].

Probiotic supplementation has been shown to positively influence the management of Escherichia coli infections in poultry through various mechanisms. For instance, the dietary inclusion of Lactobacillus acidophilus has been demonstrated to enhance growth performance, intestinal health, and survival rates in broilers challenged with *E. coli*. Additionally, supplementation with *Lacticaseibacillus rhamnosus* GG (LGG) significantly reduced the adhesion of *E. coli* to chicken intestinal epithelial cells, thereby mitigating infection severity. Furthermore, the use of competitive exclusion products, such as probiotics, has been effective in controlling the excretion and transmission of extended-spectrum beta-lactamase (ESBL)-producing *E. coli* in broiler chickens, suggesting a potential strategy for reducing antimicrobial resistance spread [44,45,46,47].

In addition to monitoring antibiotic resistance, effective prevention and control strategies are crucial in managing health risks in ostrich farming. Implementing robust biosecurity measures, such as controlling the movement of animals, improving sanitation practices, and ensuring proper waste disposal, can significantly reduce the transmission of infectious agents. Furthermore, enhancing environmental management, including reducing overcrowding and optimizing ventilation, can lower stress levels in ostriches, making them less susceptible to infections. Vaccination programs against common pathogens should also be considered as part of a comprehensive health management plan. Regular health checks and early detection of infections can enable timely interventions, reducing the likelihood of severe outbreaks. In addition, educating farmers on the importance of proper nutrition and management practices can further minimize the risk of disease and improve overall flock health.

## 4. Conclusions

This case highlights the significant challenges posed by multidrug-resistant *Escherichia coli* in ostrich farming, a rapidly growing sector with increasing concerns regarding animal health and food safety. The isolation of a resistant strain underscores the urgent need for alternative treatment strategies to combat infections caused by resistant pathogens. It is crucial to implement more stringent antimicrobial stewardship and develop more effective management practices, including improved farming conditions and better biosecurity measures, to mitigate the risks of pathogen transmission and antimicrobial resistance.

Furthermore, the potential for zoonotic transmission and cross-species pathogenicity of *E. coli* necessitates ongoing research into the genetic adaptability of avian pathogenic strains and their broader implications for both animal and human health. Considering the growing concerns over antimicrobial resistance, alternative therapeutic approaches, such as bacteriophage, probiotic and vaccination, should be explored to offer sustainable solutions for managing *E. coli* infections in ostriches and other poultry species.

## Data Availability

The original contributions presented in this study are included in the article. Further inquiries can be directed to the corresponding author.

## References

[1] Wei S., Yang Y., Tian W., Liu M., Yin S., Li J. (2020). Synergistic Activity of Fluoroquinolones Combining with Artesunate Against Multidrug-Resistant *Escherichia coli*. Microb. Drug Resist..

[2] Laxminarayan R., Duse A., Wattal C., Zaidi A.K., Wertheim H.F., Sumpradit N., Vlieghe E., Hara G.L., Gould I.M., Goossens H. (2013). Antibiotic Resistance—The Need for Global Solutions. Lancet Infect. Dis..

[3] Falagas M.E., Koletsi P.K., Bliziotis I.A. (2006). The Diversity of Definitions of Multidrug-Resistant (MDR) and Pandrug-Resistant (PDR) Acinetobacter baumannii and Pseudomonas aeruginosa. J. Med. Microbiol..

[4] Chirila F., Tabaran A., Fit N., Nadas G., Mihaiu M., Tabaran F., Catoi C., Reget O., Dan S. (2017). Concerning Increase in Antimicrobial Resistance in Shiga Toxin-Producing *Escherichia coli* Isolated from Young Animals during 1980–2016. Microbes Environ..

[5] Javed M.U., Hayat M.T., Mukhtar H., Imre K. (2023). CRISPR-Cas9 System: A Prospective Pathway toward Combatting Antibiotic Resistance. Antibiotics.

[6] Asmaa M.A., Nasr S.A.E., Ali M.M., Elshater M.A.H. (2016). Prevalence of *Escherichia coli* and Salmonella species in ostrich farms in Egypt. IOSR J. Environ. Sci. Toxicol. Food Technol..

[7] Niculae M., Vicsai M.K., Coroian I.M., Codea R., Ionuț I., Popescu S., Aurori M., Reget O., Blaga R., Tăbăran A. (2024). Antimicrobial resistance in faecal *Escherichia coli* from farmed ostrich (*Struthio camelus*). Rev. Română Med. Vet..

[8] Yehia N., Salem H.M., Mahmmod Y., Said D., Samir M., Abdel Mawgod S., Sorour H.K., AbdelRahman M.A.A., Selim S., Saad A.M. (2023). Common viral and bacterial avian respiratory infections: An updated review. Poult. Sci..

[9] Roth N., Käsbohrer A., Mayrhofer S., Zitz U., Hofacre C., Domig K.J. (2019). The application of antibiotics in broiler production and the resulting antibiotic resistance in *Escherichia coli*: A global overview. Poult. Sci..

[10] Yousef H.M.Y., Hashad M.E., Osman K.M., Alatfeehy N.M., Hassan W.M.M., Elebeedy L.A., Salem H.M., Shami A., Al-Saeed F.A., El-Saadony M.T. (2023). Surveillance of *Escherichia coli* in different types of chicken and duck hatcheries: One health outlook. Poult. Sci..

[11] Horbańczuk J., Tomasik C., Cooper R.G. (2008). Ostrich Farming in Poland—Its History and Current Situation after Accession to the European Union. Avian Biol. Res..

[12] Knöbl T., Baccaro M.R., Moreno A.M., Gomes T.A.T., Vieira M.A.M., Ferreira C.S.A., Ferreira A.J.P. (2001). Virulence properties of *Escherichia coli* isolated from ostriches with respiratory disease. Vet. Microbiol..

[13] Zhang H.L., Wu S.L., Fu J.L., Jiang H.X., Ding H.Z. (2021). Research Note: Epidemiological cutoff values and acquired resistance mechanisms of three veterinary antibiotics against *Escherichia coli* from chicken respiratory tract infections. Poult. Sci..

[14] Soares B.D., de Brito K.C.T., Grassotti T.T., Filho H.C.K., de Camargo T.C.L., Carvalho D., Dorneles I.C., Otutumi L.K., Cavalli L.S., de Brito B.G. (2021). Respiratory microbiota of healthy broilers can act as reservoirs for multidrug-resistant *Escherichia coli*. Comp. Immunol. Microbiol. Infect. Dis..

[15] Zaki R.S., Elbarbary N.K., Mahmoud M.A., Bekhit M.M., Salem M.M., Darweish M., Fotouh A. (2024). Avian pathogenic *Escherichia coli* and ostriches: A deep dive into pathological and microbiological investigation. Am. J. Vet. Res..

[16] Amani F., Hashemitabar G., Ghaniei A., Farzin H. (2020). Antimicrobial resistance and virulence genes in the *Escherichia coli* isolates obtained from ostrich. Trop. Anim. Health Prod..

[17] Poulsen L.L., Bisgaard M., Jørgensen S.L., Dideriksen T., Pedersen J.R., Christensen H. (2018). Characterization of *Escherichia coli* causing cellulitis in broilers. Vet. Microbiol..

[18] Verwoerd D.J. (2000). Ostrich Diseases. Rev. Sci. Tech. Int. Off. Epizoot..

[19] Buergelt C.D. (2000). Diseases of Ostriches and Other Ratites. J. Wildl. Dis..

[20] Samour J.H. (2021). Avian Medicine.

[21] Benameur Q., Gervasi T., Dahloum L., Rechidi-Sidhoum N., Boutaiba Benklaouz M., Yakubu A. (2023). Multidrug-resistant *Escherichia coli* isolated from cleaned and disinfected poultry houses prior to day-old chick placement. J. Environ. Qual..

[22] Glisson M.J. (1998). Bacterial respiratory disease of poultry. Poult. Sci..

[23] Kathayat D., Lokesh D., Ranjit S., Rajashekara G. (2021). Avian Pathogenic *Escherichia coli* (APEC): An Overview of Virulence and Pathogenesis Factors, Zoonotic Potential, and Control Strategies. Pathogens.

[24] Dho-Moulin M., Fairbrother J.M. (1999). Avian Pathogenic *Escherichia coli* (APEC). Vet. Res..

[25] Hedley J. (2020). BSAVA Small Animal Formulary, Part B: Exotic Pets.

[26] Finkelstein M.E., Doak D.F., George D., Burnett L.J., Brandt J., Church M., Grantham J., Smith D.R. (2012). Lead poisoning and the deceptive recovery of the critically endangered California condor. Proc. Natl. Acad. Sci. USA.

[27] Grúz A., Déri J., Szemerédy G., Szabó K., Kormos É., Bartha A., Lehel J., Budai P. (2018). Monitoring of heavy metal burden in wild birds at eastern/north-eastern part of Hungary. Environ. Sci. Pollut. Res..

[28] Pain D.J., Mateo R., Green R.E. (2019). Effects of lead from ammunition on birds and other wildlife: A review and update. Ambio.

[29] Saha O., Hoque M.N., Islam O.K., Rahaman M.M., Sultana M., Hossain M.A. (2020). Multidrug-Resistant Avian Pathogenic *Escherichia coli* Strains and Association of Their Virulence Genes in Bangladesh. Microorganisms.

[30] Fodor I., Popa V., Groza I., Catana N. (2012). Molecular Typing of Avian Pathogenic *Escherichia coli* (APEC) Using Multiprimer PCR. Sci. Res. Essays.

[31] Clinical and Laboratory Standards Institute (2020). Performance Standards for Antimicrobial Susceptibility Testing.

[32] Magiorakos A.P., Srinivasan A., Carey R.B., Carmeli Y., Falagas M.E., Giske C.G., Harbarth S., Hindler J.F., Kahlmeter G., Olsson-Liljequist B. (2012). Multidrug-resistant, extensively drug-resistant and pandrug-resistant bacteria: An international expert proposal for interim standard definitions for acquired resistance. Clin. Microbiol. Infect..

[33] Keokilwe L., Olivier A., Burger W.P., Joubert H., Venter E.H., Morar-Leather D. (2015). Bacterial Enteritis in Ostrich (*Struthio camelus*) Chicks in the Western Cape Province, South Africa. Poult. Sci..

[34] Cooper R.G. (2005). Bacterial, Fungal, and Parasitic Infections in the Ostrich (*Struthio camelus* var. *domesticus*). Anim. Sci. J..

[35] Chong S.M., Douay G., Ahmad A.A., Yeong C.Y., Heng Y., Oh P.Y., Chua D., Xie S. (2023). *Enterococcus casseliflavus* Infection in a Captive Southern Cassowary (*Casuarius casuarius*): Clinical and Post-Mortem Findings. J. Vet. Med. Sci..

[36] Hussein A.H.M., Ghanem I.A.I., Eid A.A.M., Ali M.A., Sherwood J.S., Li G., Nolan L.K., Logue C.M. (2013). Molecular and phenotypic characterization of *Escherichia coli* isolated from broiler chicken flocks in Egypt. Avian Dis..

[37] Abd El Tawab A.A., Maarouf A.A.A., Abd El Al S.A., El Hofy F.I., El Mougy E.E.A. (2014). Detection of some virulence genes of avian pathogenic *Escherichia coli* by polymerase chain reaction. Benha Vet. Med. J..

[38] Mellata M. (2013). Human and avian extraintestinal pathogenic *Escherichia coli*: Infections, zoonotic risks, and antibiotic resistance trends. Foodborne Pathog. Dis..

[39] Youssef A., Afifi R. (2017). Zoonotic potential of Salmonella and *Escherichia coli* isolated from ostrich eggs of a flock in a recreational park. Hum. Vet. Med..

[40] Ščerbová J., Lauková A. (2016). *Escherichia coli* strains from ostriches and their sensitivity to antimicrobial substances. Pol. J. Vet. Sci..

[41] Văduva D.B., Velimirovici D.E., Vaduva M.M., Stanga L., Petrescu H.P., Rada M.P., Cipu D., Vaduva B.M., Rădulescu M. (2018). Phenotypic Study and Sensitivity to Anti-Infective Chemotherapy of Bacterial Strains Isolated from Cutaneous-Mucosal Infections. Mater. Plast..

[42] Huff W.E., Huff G.R., Rath N.C., Balog J.M., Xie H., Moore P.A., Donoghue A.M. (2002). Prevention of *Escherichia coli* respiratory infection in broiler chickens with bacteriophage (SPR02). Poult. Sci..

[43] El-Tahawy A.O., Said A.A., Shams G.A., Hassan H.M., Hassan A.M., Amer S.A., El-Nabtity S.M. (2022). Evaluation of cefquinome’s efficacy in controlling avian colibacillosis and detection of its residues using high-performance liquid chromatography (HPLC). Saudi J. Biol. Sci..

[44] El-Mawgoud A.I.A., El-Nahass E.S., Shany S.A.S., El-Sawah A.A., Dahshan A.M., Nasef S.A., Ali A. (2020). Efficacy of Live Attenuated Vaccine and Commercially Available Lectin Against Avian Pathogenic *E. coli* Infection in Broiler Chickens. Vet. Sci..

[45] Ebrahem A.F., El-Demerdash A.S., Orady R.M., Nabil N.M. (2024). Modulatory Effect of Competitive Exclusion on the Transmission of ESBL E. coli in Chickens. Probiotics Antimicrob. Proteins.

[46] Guo M., Zhang C., Zhang C., Zhang X., Wu Y. (2021). *Lacticaseibacillus rhamnosus* Reduces the Pathogenicity of *Escherichia coli* in Chickens. Front. Microbiol..

[47] Wu Z., Yang K., Zhang A., Chang W., Zheng A., Chen Z., Cai H., Liu G. (2021). Effects of *Lactobacillus acidophilus* on the Growth Performance, Immune Response, and Intestinal Barrier Function of Broiler Chickens Challenged with *Escherichia coli* O157. Poult. Sci..

